# Synergistic Anti-Angiogenic Effects Using Peptide-Based Combinatorial Delivery of siRNAs Targeting VEGFA, VEGFR1, and Endoglin Genes

**DOI:** 10.3390/pharmaceutics11060261

**Published:** 2019-06-06

**Authors:** Anna A. Egorova, Sofia V. Shtykalova, Marianna A. Maretina, Dmitry I. Sokolov, Sergei A. Selkov, Vladislav S. Baranov, Anton V. Kiselev

**Affiliations:** 1 D.O. Ott Research Institute of Obstetrics, Gynecology and Reproductology, 199034 Saint-Petersburg, Russia; egorova_anna@yahoo.com (A.A.E.); marianna0204@gmail.com (M.A.M.); falcojugger@yandex.ru (D.I.S.); selkovsa@mail.ru (S.A.S.); baranov@vb2475.spb.edu (V.S.B.); 2 Department of Genetics and Biotechnology, Saint-Petersburg State University, 199034 Saint-Petersburg, Russia; sofia.shtykalova@gmail.com

**Keywords:** VEGFA, VEGFR1, endoglin, siRNA delivery, peptide, angiogenesis, gene silencing, migration, proliferation, endothelial cells

## Abstract

Angiogenesis is a process of new blood vessel formation, which plays a significant role in carcinogenesis and the development of diseases associated with pathological neovascularization. An important role in the regulation of angiogenesis belongs to several key pathways such as VEGF-pathways, TGF-β-pathways, and some others. Introduction of small interfering RNA (siRNA) against genes of pro-angogenic factors is a promising strategy for the therapeutic suppression of angiogenesis. These siRNA molecules need to be specifically delivered into endothelial cells, and non-viral carriers modified with cellular receptor ligands can be proposed as perspective delivery systems for anti-angiogenic therapy purposes. Here we used modular peptide carrier L1, containing a ligand for the CXCR4 receptor, for the delivery of siRNAs targeting expression of VEGFA, VEGFR1 and endoglin genes. Transfection properties of siRNA/L1 polyplexes were studied in CXCR4-positive breast cancer cells MDA-MB-231 and endothelial cells EA.Hy926. We have demonstrated the efficient down-regulation of endothelial cells migration and proliferation by anti-VEGFA, anti-VEGFR1, and anti-endoglin siRNA-induced silencing. It was found that the efficiency of anti-angiogenic treatment can be synergistically improved via the combinatorial delivery of anti-VEGFA and anti-VEGFR1 siRNAs. Thus, this approach can be useful for the development of therapeutic angiogenesis inhibition.

## 1. Introduction

Angiogenesis is the process of the formation of a new capillary network. A crucial role in the angiogenesis regulation belongs to mechanisms associated with endothelial cells, which retain their ability to divide in the adult organism [1]. Angiogenesis includes all phases of new blood vessel growth: proliferation and migration of endothelial cells, the formation of a capillary tube, and the remodulation of vascular network in organs [2,3]. The balanced functioning of this system is very important, since either the excessive vessel formation or their insufficient development leads to serious diseases. For example, intensive angiogenesis contributes to tumor growth where the formation of a branched vascular network in the tumor leads to an increase in their growth and further metastasis [4]. Therefore, approaches for the down-regulation of angiogenesis are used as elements for therapy against these diseases. Also, endothelial cells, being genetically stable, are less likely to develop drug resistance in comparison with tumor ones [5]. Taken together, antitumor agents targeting endothelial cells are supposed to be more effective than drugs targeting tumor cells. The inhibition of endothelial cell proliferation and migration may lead to the lack of structural support for tumor cells resulting in the disassembly of tumor tissues and can be used for the treatment of cancers and tumor-like diseases [6,7].

A key role in angiogenesis belongs to VEGFA (the vascular endothelial growth factor A). Its level is elevated in tissues with intensive angiogenesis and its receptors are predominantly expressed on the endothelial cells of blood vessels nearby [8,9]. VEGFA signaling has a direct impact on endothelial cells growth in vitro [10]. It prevents apoptosis of endothelial cells in vitro by inducing the expression of anti-apoptotic proteins Bcl-2 and A1 [11]. In newborn mice the suppression of VEGFA gene expression leads to apoptosis of endothelial cells in a large number of blood vessels [12]. Hypoxia is an upregulating factor for VEGFA gene expression and signaling [13,14]. For example, solid tumors under hypoxia can stimulate the production of an increased amount of VEGFA [15]. This creates conditions for the intensive development of vascular network in the growing tumors. VEGFA signaling functions through two receptor tyrosine kinases of similar structure: VEGFR1 and VEGFR2. VEGFR2 is considered to be the primary VEGFA receptor that runs angiogenesis, while VEGFA most strongly binds to the VEGFR1 receptor. VEGFR1 gene knockout mice die on the ninth day of prenatal development from disorganization and excessive growth of blood vessels. This showed that in early embryogenesis, VEGFR1 functions mainly as a decoy receptor that sequesters excess VEGFA [16]. Nevertheless, a positive modulation of angiogenesis by VEGFR-1 has been demonstrated in adults. For example, suppression of VEGFR1 led to defects in neovascularization of the eye [17]. Also, VEGFR1 expression is high in many human cancers [16,18]. VEGFR1 is suggested to serve as an alternative angiogenic pathway in the case when VEGFA is inhibited, acting in conjunction with VEGFB and PlGF ligands [19]. It gives the opportunity for VEGFR1-based inhibition of angiogenesis alternatively to the VEGFA/VEGFR2 pathway. One more alternative and promising target for the inhibition of angiogenesis is endoglin. Its expression is greatly increased in endothelial cells of blood vessels and surrounding tumors [20]. Endoglin (ENG or CD105) is a co-receptor for transforming growth factor-β (TGF-β), which participates in activating a complex signaling pathway and thus mediates the proliferation, migration, and adhesion of endothelial cells [21]. Mice with a fully inactivated endoglin die during prenatal development due to cardiac abnormalities and defects in the formation of the vascular network; their vessels stop growing and do not penetrate into the yolk sac [22]. Homozygous knockout the endoglin gene mutations in humans are also lethal. Heterozygous endoglin mutations cause hereditary hemorrhagic telangiectasia 1, which is characterized by the fragility and instability of small vessels [23]. Endoglin is preferentially expressed in the angiogenic endothelium of solid tumors, and was found to be a marker of activated endothelial cells [24]. Recently it was suggested as a promising target for antivasculogenic therapy [25,26].

A possible way for angiogenesis targeting may be the use of a gene therapy approach where small interfering RNA (siRNA) is introduced into endothelial cells with the aim to specifically inhibit the pro-angiogenic gene expression. The application of siRNA for the specific suppression of endogenous genes in cells was successfully realized by Elbashir et al. [27].

One of the most important barriers to the application of RNAi remains the necessity to create an effective siRNA delivery system. The delivery system has to provide siRNA-targeted delivery into cells, protect it from nucleases degradation, and release siRNA for its activity in the cytosol [28]. To achieve nucleic-acid-targeted delivery to endothelial cells, we and others previously suggested a new ligand-receptor pair SDF1/CXCR4 [29,30,31,32]. SDF1 (stromal cell-derived factor-1) is a ligand for the CXCR4 (chemokine receptor type 4) expressed in the endothelium of angiogenic vessels [33]. Moreover, SDF1 plays an important role during neoangiogenesis by being a main recruiter of endothelial progenitor cells [34]. The targeted delivery via CXCR4 was achieved in our previous studies by using modular peptide carriers modified with ligand derived from the N-terminus of SDF1 [31,35,36,37,38,39]. The developed carriers were based on cationic cysteine-flanked cross-linking peptides that can effectively bind and protect DNA and RNA fromnuclease degradation [35,36,37]. The anti-VEGFA siRNA-peptide polyplexes demonstrated an efficient inhibition of VEGFA expression in endothelial cells in vitro [37,39].Achieved VEGFA gene silencing by means of RNAi resulted in a significant decrease of VEGFA protein production and the rate of endothelial cell migration. Polyplexes were formed by anti-VEGFA siRNA and the most efficient peptide carrier L1 was tested using an in vivo treatment of endometriosis in a rat subcutaneous model [38]. Significantinhibition of endometriotic implants growth (55–60%) and a two-fold decrease in VEGFA gene expression were demonstrated. Anti-angiogenic effect of the polyplexes also was confirmed via immunohistochemical characterization of the endometriotic implants.

In the present study, we used L1 peptide-based polyplexes bearing siRNA against VEGFA, VEGFR1, and endoglin for the targeted suppression of angiogenesis in endothelial cells. Proliferation and migration of the transfected cells was evaluated. We analyzed the effects of VEGFA, VEGFR1, and endoglin gene silencing, either alone or in combination. Here, the tested hypothesis was that the combinatorial siRNA silencing of several angiogenic pathways may be more efficient than single gene knockdown and could result in synergistic anti-angiogenic effects in endothelial cells.

## 2. Materials and Methods

### 2.1. Cell Lines

GFP-expressing human breast cancer cell line MDA-MB 231 was kindly provided by Prof. Jessica Rosenholm, Abo Academy University, Turku, Finland. The cell line was maintained under mycoplasma-free conditions as described previously [40].

Endothelial cells EA.Hy926 (hybridoma of primary HUVEC (human umbilical vein endothelial cells) and A549 cells (human lung adenocarcinoma)) were kindly gifted by Dr. Cora-Jean C. Edgell from the University of North Carolina, USA. This cell line reproduces the main morphological, phenotypical and functional features of the endothelium [41]. The EA.Hy926 cells were maintained under mycoplasma-free conditions as described previously [36].

### 2.2. Peptide Synthesis and Design

L1 peptide carrier was synthesized using NPF Verta, LLC (SaintPetersburg, Russia), and stored desiccated at −20 °C. Before use, the peptide carrier was dissolved in 0.1% TFA at 2 mg/mL. The peptide purity was determined using high-performance liquid chromatography, and found to be in the range of 90–95%. L1 peptide consists of the KPVSLSYRSPSRFFESH motif connected with a DNA-binding sequence (CHRRRRRRHC) via two ε-aminocaproic acids (Ahx) [36].

### 2.3. siRNA Preparation of Peptide/siRNA Complexes

The sense strand of anti-VEGFA siRNA 5′-GCG GAU CAA ACC UCA CCA Att-3′ targets human VEGFA mRNA [42]. The sense strand of anti-VEGFR1 siRNA 5′-GGC CAA GAU UUG CAG AAC Utt-3′ targets human VEGFR1 mRNA [43]. The sense strand of anti-endoglin siRNA 5′-CGG UGA CGG UGA AGG UGG AAC UGA G-3′targets human endoglin mRNA [44]. The sense strand of anti-GFP siRNA 5′-CAA GCU GAC CCU GAA GUU Ctt-3′ targets GFP mRNA [45]. A non-silencing siRNA 5′-UUC UCC GAA CGU GUC ACG U- 3′served as a mock siRNA [46]. siRNAs were purchased from Syntol JSC, Moscow, Russia. siRNA/peptide complexes were prepared at 8 to 1 and 16 to 1 N/P ratios (peptide nitrogen/RNA phosphorus ratio). All positively charged amino acids were taken into account for the calculation of N/P charge ratios. The appropriate volume of the peptide carrier (2 mg/mL) was added to the siRNA solution (100 μg/mL) in Hepes-buffered mannitol (HBM) (5% *w*/*v* mannitol, 5 mM Hepes, pH 7.5) and vortexed. Then, thepolyplexes were allowed to stand at room temperature for 2 h.

X-tremeGENE liposomal transfection reagent (Roche, Mannheim, Germany) was used as a control siRNA carrier according to the manufacturer recommendations.

### 2.4. Cytotoxicity Assay

A total of 0.6 × 10^4^ MDA-MB-231 and ЕА.Hy926 cells (at the low density) and 2.5 × 10^4^ ЕА.Hy926 cells (at the high density) were seeded in 96-well plates and incubated overnight. The cytotoxicity of peptide/siRNA complexes was evaluated using Alamar blue assay (Invitrogen, Eugene, OR, USA) for cell viability after 16 h of incubation. The fluorescence was recorded on a Wallac 1420D scanning multilabel counter (Thermo Fisher Scientific Oy, Vantaa, Finland) with an excitation wavelength at 544 nm and emission wavelength at 590 nm. The relative fluorescence intensity was counted according to (F−Ff)/(Fb−Ff) × 100%, where Fb is the fluorescence intensity in untreated control and Ff is the fluorescence intensity without cells. The results are presented as mean± S.E.M of the means obtained from three independent experiments with three samples.

### 2.5. siRNA Transfer to MDA-MB-231 Cells

Transfection experiments were performed in triplicate. A total of 2.5 × 10^4^ cells was seeded in 24-well plates and incubated overnight. Before transfection, the cell culture medium was replaced with medium without FBS.Anti-GFP siRNA and mock siRNA complexes were added and incubated with cells for 2.5 h. The final concentration of siRNA was 200 nM in each well and the volume of medium was 250 µL. After incubation in 1000 µL of normal culture medium for the next 48 h, cells were washed cells by 1× PBS (pH 7.2) and permeabilized with the reporter cell lysis buffer (25 mM Gly-Gly, 15 mM MgSO_4_, 4 mM EGTA, 1 mM DTT, 1 mM PMSF; pH 7.8). GFP fluorescence in the cell extracts was measured with a Wallac 1420D scanning multilabel counter (Thermo Fisher Scientific Oy, Vantaa, Finland) at excitation wavelength of 485 nm and emission wavelength of 535 nm. The GFP fluorescence level was normalized by the total protein concentration of the cell extracts, measured using Bradford reagent (Helicon, Moscow, Russia). The data are shown as mean ± S.E.M of the means obtained from three independent experiments with three samples. Visual appearance of MDA-MB-231 cells after the transfection was registered using a Leica DM 2500 microscope (Wetzlar, Germany) with a Leica DFC345 FX camera.

### 2.6. siRNA Transfer to ЕА.Hy926 Cells

Transfection experiments in ЕА.Hy926 cells were performed in duplicates. The cells (15 × 10^4^) were seeded in 24-well plates and incubated overnight. A fully supplemented cell culture medium was aspirated and replaced with medium without FBS just before the addition of siRNA complexes, followed by incubation for 4 h. The final concentration of siRNA was 200 nM per well in 1000 µL of medium. After incubation in a fully supplemented cell culture medium for the next 48 h, cells were taken for RNA extraction.

### 2.7. Quantitative RT-PCR

Total RNA extraction and quantitative real-time PCR analysis was performed as previously described [36,38]. The following primers were used: VEGFR1 forward primer 5′-GAGCTAAAA ATCTTGACCCACATTG-3′, reverse primer 5′-CAGTATTCAACAATCACCATCAGAG-3′; endoglin forward primer 5′-TGGTACATCTACTCGCACACGC-3′, reverse primer 5′-GGCTATGCCATGCTG CTGGTGG-3′; and endogenous reference gene β-actin was detected using forward 5′-TGCCGACAGGATGCAGAAG-3′, reverse primer 5′-GCCGATCCACACGGAGTACT-3′. The samples were measured three times and a final result was inferred by averaging the data. The values are presented as mean ± S.E.M of the means obtained from three independent experiments.

### 2.8. Scratch Migration Assay

The ЕА.Hy926 cells migration study of was performed as described previously [37,39]. siRNA/L1 complexes were prepared as described above at N/P ratios of 8/1 and 16/1 in quadruplicates (siRNA concentration was 200 and 100 nM). Also, we used a combination of different siRNA in the complexes. Stained cells were photographed using an AxioObserver Z1 microscope (Carl Zeiss, Jena, Germany). Three random fields were registered. EA.Hy926 cell migration during the wound repair was analyzed using ImagePro Plus 6.0 software (Media Cybernetics, Bethesda, MD, USA). Number of cells (n) that migrated to the wound area was counted. Cell density (ρ) was counted in area of 17,000 µm^2^. Relative number of migrated cells was computed by (n/n’)*(ρ’/ρ), where n’ is number of migrated cells in untreated control and ρ’ is the cell density in the untreated control. The results are presented as mean± S.E.M of the means obtained from five independent experiments with four samples.

### 2.9. Proliferation Assays

For the сell proliferation study, 0.6 × 10^4^ ЕА.Hy926 cells in 100 µL DMEM-F12 medium per well were plated in a 96-well plate. siRNA/L1 complexes were prepared as described above at N/P ratios of 8/1 and 16/1 in quadruplicates (siRNA concentration was 200 and 100 nM). Also, we used a combination of different siRNA in the complexes. The cell culture medium was replaced with 50 µL of medium without FBS. siRNA/peptide complexes were added and incubated with cells for 2.5 h.Then, the cell culture medium was replaced with 100 µL of medium containing 2.5% FBS. After 72 h incubation, the cell proliferation was analyzed using Alamar blue or crystal violet staining. In the case of AlamarBlue assay, cells were incubated in a normal culture medium with 10% Alamar blue for 2 h. The fluorescence was recorded on a Wallac 1420D scanning multilabel counter (Thermo Fisher Scientific Oy, Vantaa, Finland) with an excitation wavelength at 544 nm and emission wavelength at 590 nm.In the case of crystal violet analysis, cells were stained in 100 µL of 0.2% crystal violet in 5% methanol and were then dried. Cells were dissolved in 100 µL of 50% acetic acid per well for 5 min.Absorbance was measured at 540 nm and at 630 nm. The 630 nm values were subtracted from the 450 nm values to correct for optical imperfections in the microplate. The results are shown as mean±S.E.M of the means obtained from five independent experiments with four samples.

### 2.10. Statistical Analysis

Statistical analysis was carried out using the Student *t*-test with the GraphPad Prism 6 software package (GraphPad Prism Inc., San Diego, CA, USA). Statistical significance was defined as * *p* < 0.05, ** *p* < 0.01, and *** *p* < 0.001.

## 3. Results and Discussion

RNAi targeting of different angiogenic pathways gives an opportunity for the combinatorial anti-angiogenic treatment of tumor diseases. The therapeutic RNAi can stop the growth of tumor vessels andeventually result in the disassembly of tumor tissues [47]. Here, we targeted three pathways by means of siRNA-mediated down-regulation of VEGFA, VEGFR1, and endoglin molecules. For the siRNA delivery, a previously developed original vector L1, targeting of the CXCR4 receptor was used. MDA-MB 231 and ЕА.Hy926 cell lines, used for the study, were shown to express CXCR4 on their surface [37,48,49]. CXCR4 is a promising receptor to be targeted for tumor-vasculature-specific delivery due to a previously found dependence between the receptor density on target cells and the efficiency of gene delivery by means of CXCR4-targeted vehicles [29,30,31]. This feature may additionally minimize possible off-target effects mediated by siRNA delivery in vivo via the CXCR4 receptor.

### 3.1. Cytotoxicity Evaluation of L1 Peptide/siRNA Complexes

**Figure 1:**
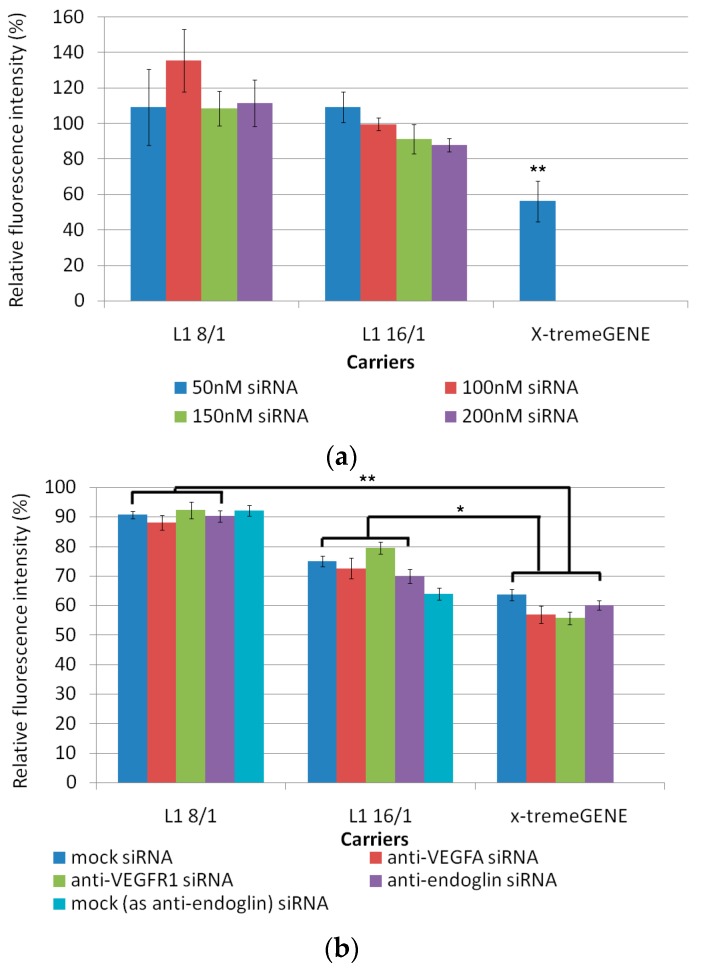

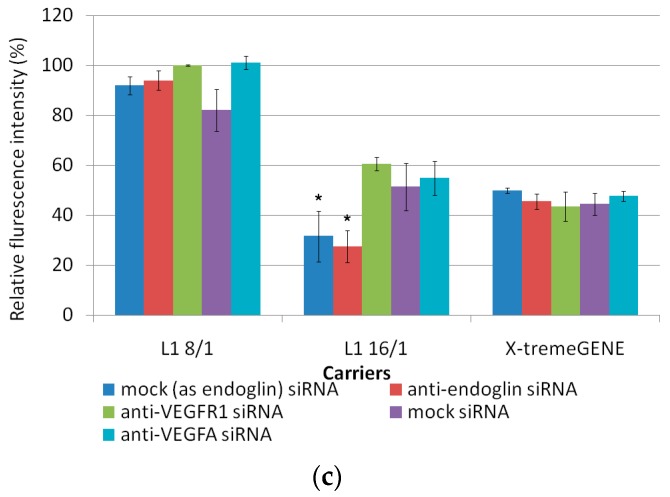
Cytotoxicity evaluation of the siRNA-polyplexes in MDA-MB-231 cells at the low density (**a**), ЕА.Hy926 cells at the low density (**b**), and ЕА.Hy926 cells at the high density (**c**). * *p* < 0.05, ** *p* < 0.01 when compared with X-tremeGENE-lipoplexes.

Cytoxicity studies are critical for establishing of the potential of nanocarrier systems for gene therapy [50]. Cytotoxicity of the siRNA-polyplexes at two charge ratios (8/1 and 16/1) was determined using an Alamar Blue assay in MDA-MB-231 and ЕА.Hy926 cell lines (Figure 1). ЕА.Hy926 cells were seeded at high (25,000 per well) and low (6000 per well) densities according to the requirements of the migration and the proliferation analysis protocols, respectively. The low density of cells may affect results of the proliferation test after the transfection. That is why the cytotoxicity of siRNA-polyplexes in model MDA-MB-231 GFP-expressing cells, seeded at low density, was studied to choose a non-toxic siRNA-polyplexes concentration for the subsequent transfection and proliferation studies. Cytotoxicity was determined for L1/siRNA polyplexes and X-tremeGENE/siRNA lipoplexes. In the case of MDA-MB-231 cells, we used different anti-GFP siRNA concentrations (50, 100, 150, and 200 nM). We found that L1/siRNA-polyplexes at the all siRNA concentrations showed significantly lower cell toxicity than the X-tremeGENE/siRNA lipoplexes (Figure 1a). The relative fluorescence intensity after cell incubation with these polyplexes was not less than 80% to that of intact cells. These results supposed that the studied L1/siRNA-polyplexes were not involved in MDA-MB-231 cell damage and could be used in subsequent studies. In experiments with ЕА.Hy926 cells, we used anti-VEGFA, anti-VEGFR1, anti-endoglin, and mock siRNAs to evaluate the impact of a cationic peptide carrier on the cytotoxicity of siRNA-polyplexes. Studies with ЕА.Hy926 cells were conducted using 200 nM of siRNA, which was referred to our previous research on cells with high density [37]. Due to the fact that anti-endoglin siRNA has a higher molecular weight, the corresponding siRNA polyplexes consisted of more cationic L1 carrier. In order to exclude a contribution of cationic carrier on cellular damage in subsequent functional tests, we formulated mock siRNA-polyplexes with the amount of peptide carrier equivalent to anti-endoglin siRNA-polyplexes and used it as an additional control. In a preliminary experiment in endothelial cells at the low density, several siRNA concentrations were tested (50, 100, 150, and 200 nM), and no difference in cytotoxicity of the polyplexes was found (data not shown). Therefore, 200 nM of siRNA was used in subsequent experiments. In ЕА.Hy926 cells seeded at the low density, siRNA-polyplexes at 8/1 charge ratios were found to be non-toxic and the relative fluorescence intensity after cell incubation with these polyplexes was about 90% to that of intact cells. L1/siRNA complexes at a charge ratio of 16/1 were more toxic in comparison with 8/1 polyplexes.The relative amount of viable cells was 65–80% in comparison with that in the intact cells. However, the cytotoxicity of these polyplexes was less than that of X-tremeGENE/siRNA lipoplexes (Figure 1b).

The experiments with ЕА.Hy926 cells seeded with high density are showed in Figure 1c. The results of Alamar blue assay suggested that at N/P ratio of 8/1 the L1/siRNA complexes had no apparent cytotoxicity. The relative fluorescence intensity after cell incubation with siRNA-polyplexes at a 8/1 charge ratio was similar to that of intact cells. However, cytotoxicity was detected for L1/anti-endoglin siRNA and corresponding mock siRNA polyplexes formed at a N/P ratio of 16/1. It is known that the cytotoxicity of the polyplexes is mainly caused by their positive surface charge. The cationic nanocarriers interact with the cell membranes changing the membrane potential and porosity, thus eventually inducing inflammatory responses [51]. In our previous studies, a zeta potential of the L1-polyplexes formed at a 16/1 N/P ratio was shown to be highly positive (+35 mV) [37]. However, polyplexes with a positive zeta-potential were used to provide the higher transfection efficiency [52]. In the case of mock, anti-VEGFA, and anti-VEGFR1 siRNA L1-polyplexes at a 16/1 N/P ratio, the cytotoxicity was similar to that of corresponding X-tremeGENE-lipoplexes. Also, cytotoxicity exhibited by anti-endoglin siRNA-polyplexes formed at N/P ratio 16/1 was higher than in X-tremeGENE-lipoplexes. Thus, we decided to exclude the anti-endoglin siRNA-polyplexes from subsequent migration studies.

### 3.2. In Vitro Transfection of MDA-MB-231 Cells

**Figure 2:**
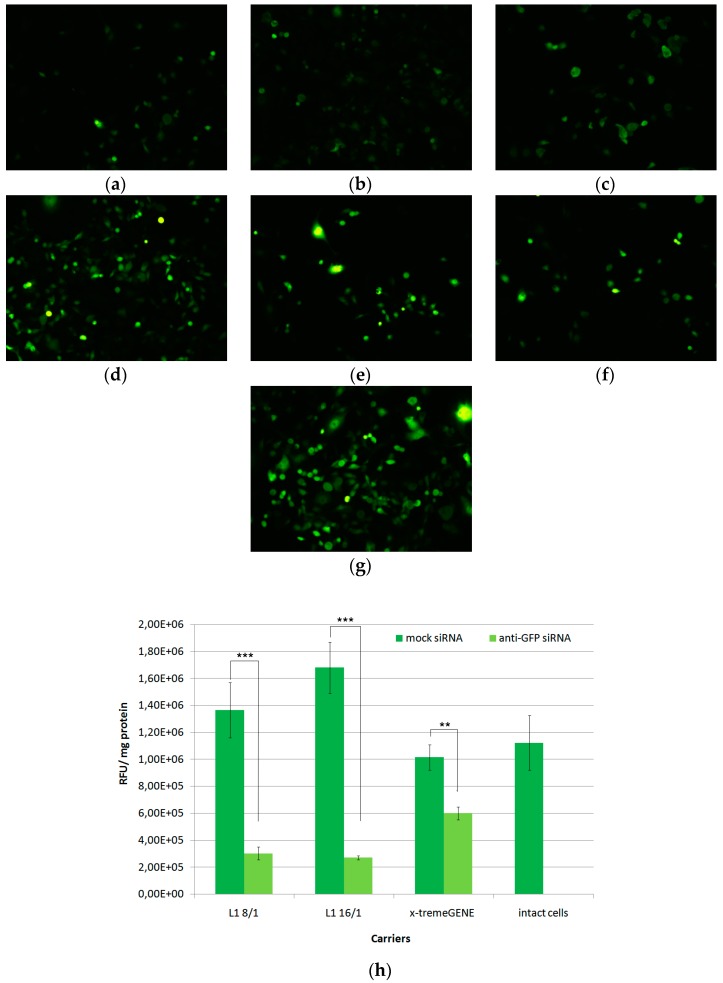
Silencing of the *GFP* gene expression after the treatment of MDA-MB-231 cells with L1/siRNA and X-tremeGENE/siRNA complexes. The visual appearance of MDA-MB-231 cells (magnification ×100) after treatment with (**a**) L1/anti-GFP siRNA (N/P 8/1),(**b**) L1/anti-GFP siRNA (N/P 16/1), (**c**) x-tremeGENE/anti-GFP siRNA, (**d**) L1/mock siRNA (N/P 8/1), (**e**) L1/mock siRNA (N/P 16/1), (**f**) x-tremeGENE/mock siRNA complexes (200 nM siRNA), and (**g**) intact cells. (**h**) Quantitative analysis of *GFP* gene expression, ** *p* < 0.01, *** *p* < 0.001 when compared with cells treated by mock siRNA-complexes.

MDA-MB-231 GFP-positive breast cancer cells were used as a convenient model for marker gene silencing in vitro. An anti-GFP siRNA transfer was performed by means of L1- and X-tremeGENE-based complexes at 8/1 and 16/1 charge ratios and with 200 nM of siRNA in 50 µL of medium (Figure 2). MDA-MB-231 cells were seeded at the low density corresponding to that in the proliferation protocol in order test whether cell density can affect the silencing efficiency. At the time of transfection, the cell growth was in log-phase. L1/mock siRNA complexes were used as a negative control and did not down-regulated GFP gene expression in comparison with non-treated cells. Meanwhile, L1/anti-GFP polyplexes caused significant decrease of GFP gene expression compared to negative control. This fact strongly confirms siRNA-induced marker gene expression silencing in the model cells. Transfection with L1/anti-GFP-siRNA complexes at N/P ratios of 8/1 and 16/1 resulted in a decrease of the relative GFP expression level of up to 22% and 17% from the intact cells, respectively. X-tremeGENE/anti-GFP-siRNA complexes also showed down-regulation of GFP gene expression up to 59%.Thus, at the low cell density conditions, we observed targeted inhibition of gene expression by means of RNAi, and the results obtained allowed us to further use RNAi in the proliferation protocol.

### 3.3. In Vitro Transfection of ЕА.Hy926 Cells

**Figure 3:**
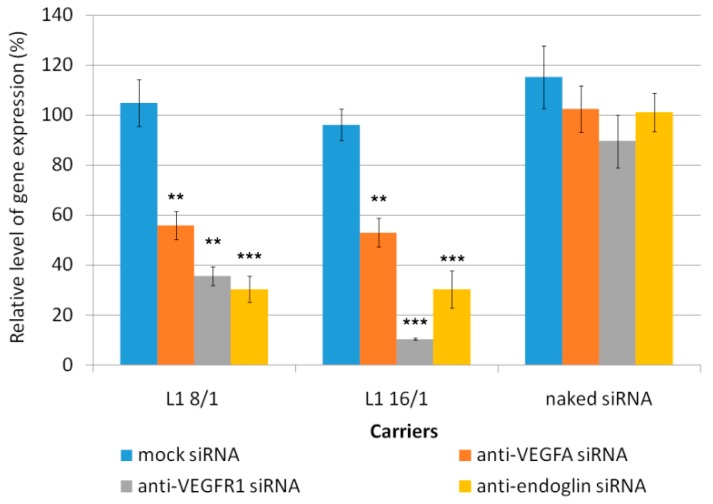
Silencing of VEGFA, VEGFR1, and endoglin gene expression after the treatment of ЕА.Hy926 cells by L1/siRNA polyplexes. ** *p* < 0.01, *** *p* < 0.001 when compared with cells treated using L1/mock siRNA polyplexes.

Effects of L1 carrier-mediated siRNA delivery on gene expression were also investigated in endothelial ЕА.Hy926 cells. The main morphological and functional characteristics of this cell line allowed us to use it as a model of vascular endothelium and to study angiogenesis down-regulation [40]. Before performing functional tests, it was necessary to prove the specificity of VEGFA, VEGFR1, and endoglin gene expression inhibition via the RNAi mechanism. Anti-VEGFA, anti-VEGFR1, anti-endoglin, and mock siRNA were used to induce the specific silencing effects on the corresponding genes expression. L1/siRNA polyplexes were formed at 8/1 and 16/1 N/P ratios. The mock siRNA-polyplexes and free siRNA were used as negative controls, which did not show down-regulation of the gene expression (Figure 3).

The cell treatment using L1/anti-VEGFA-siRNA complexes at N/P ratios of 8/1 and 16/1 resulted in a decrease of VEGFA gene expression of up to 56% and 53%, respectively. L1/anti-VEGFR1 polyplexes formed at N/P ratios of 8/1 and 16/1 demonstrated VEGFR1 knockdown of up to 36% and 11%, respectively. In the case of L1/anti-endoglin polyplexes formed at the same charge ratios down-regulated the endoglin gene expression up to 31% and 30%, respectively. The complexes with nonspecific mock siRNA did not induce any silencing compared to anti-VEGFA, anti-VEGFR1, or anti-endoglin siRNA-polyplexes. Taken together, these results suggest that the reduction in the appropriate gene expression in endothelial cells was due to a specific siRNA effect but not from the carrier toxicity.

### 3.4. Inhibition of the Endothelial Cells Migration

**Figure 4:**
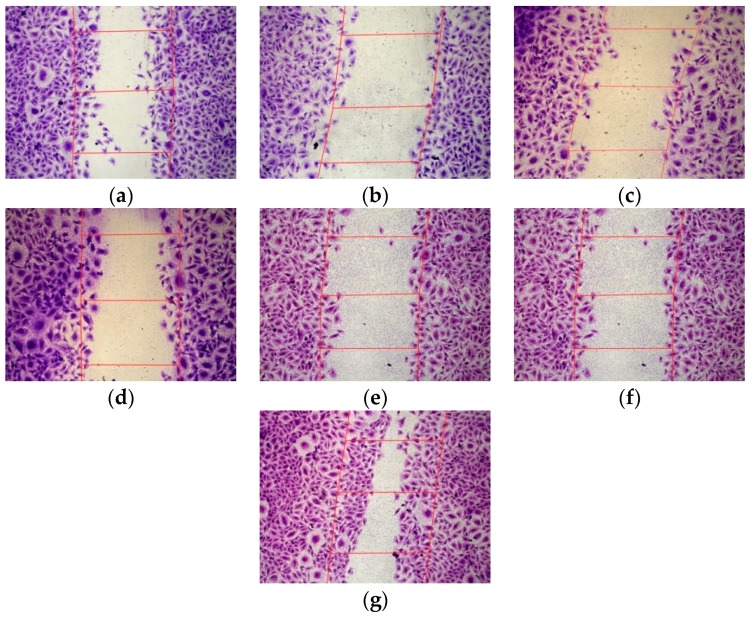
Appearance of migrated ЕА.Hy926 cells (magnification ×100) after treatment with (**a**) L1/mock siRNA,(**b**) L1/anti-VEGFA siRNA, (**c**) L1/anti-VEGFR1 siRNA, (**d**) L1/anti-endoglin siRNA, (**e**) L1/anti-VEGFA siRNA + anti-VEGFR1 siRNA, (**f**) L1/anti-VEGFA siRNA+anti-endoglin siRNA complexes (200 nM siRNA), and (**g**) intact cells.

**Figure 5:**
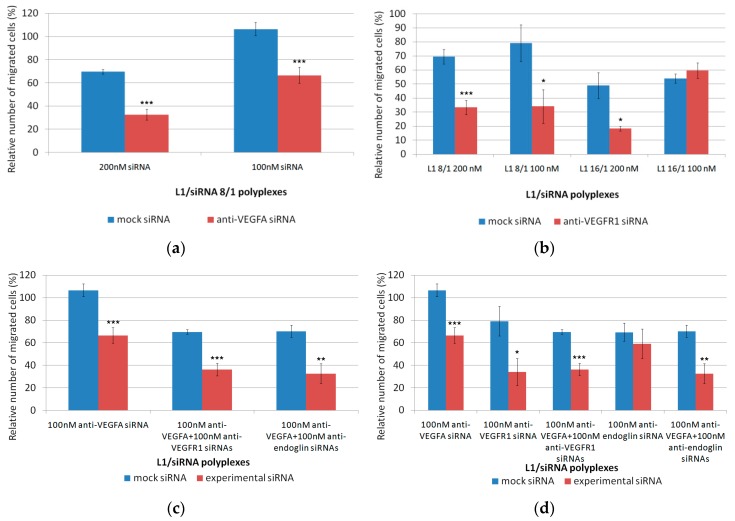
Number of migrated ЕА.Hy926 cells after treatment with (**a**) L1/anti-VEGFA siRNA, (**b**) L1/anti-VEGFR1 siRNA, (**c**) L1/ anti-endoglin siRNA, and (**d**) L1/mixed siRNA complexes. * *p* < 0.05, ** *p* < 0.01, *** *p* < 0.001 when compared with cells treated using mock siRNA.

Migration of endothelial cells is a necessary step in the formation of new blood vessels. Endothelial cells migrate from already existing blood vessels to angiogenesis foci and interact with vascular smooth muscle cells and pericytes of the new vessels [53]. Scratch assays were performed to determine how EA.Hy926 cell migration can be affected by anti-VEGFA, anti-VEGFR1, or anti-endoglin siRNA delivery. L1/mock siRNA polyplexes were used as a negative control to demonstrate the specificity of the siRNA action. EA.Hy926 cells were transfected with siRNA-bearing polyplexes with different charge ratios and siRNA concentration. The cell monolayer was damaged and the relative number of migrated cells into the cell-free area was registered (Figure 4). The number of migrated intact cells was taken as 100% (Figure 5). It should be noted that efficient VEGFA gene silencing by L1-based polyplexes bearing 200 nM of siRNA was demonstrated previously [37]. Here, we compared the VEGFA gene silencing efficacy in EA.Hy926 cells treated with 100 and 200 nM of anti-VEGFA siRNA. The L1-based polyplexes were formed at the N/P ratio of 8/1. The decrease of cell migration after polyplex treatment with 100 nM and 200 nM of siRNA was found to be to 63% and 47%, respectively, in comparison with an appropriate mock siRNA control (Figure 5a). Actually, a key role in stimulating the blood vessels formation belongs to VEGFA. For example, VEGFA stimulates the endothelial cell migration by interacting with neuropilin-1 [54]. Therefore, a decrease in the expression of the VEGFA gene eventually leads to reduction of the cell migration.

In the case of anti-VEGFR1 siRNA treatment, the complexes were formed at the N/P ratios of 8/1 and 16/1, and with siRNA concentrations of 100 nM and 200 nM (Figure 5b). Anti-VEGFR1 siRNA complexes formed at the 16/1 N/P ratio and with 100 nM of siRNA did not contribute to a decrease in the migration activity of endothelial cells in comparison with mock siRNA-polyplexes. In contrast, significant differences between anti-VEGFR1 siRNA and mock siRNA-complexes were found for the inhibition efficiency of L1 polyplexes formed at the N/P ratio of 8/1 with 100 nM or 200 nM siRNA, and the N/P ratio of 16/1 with 200 nM siRNA. A decrease of cell migration was found to be up to 43%, 47%, and 37%, respectively, compared to appropriate mock siRNA-complexes. Therefore, in most cases, a decrease in VEGFR1 gene expression led to a reduction of endothelial cell migration. Previously, it was also demonstrated that down-regulation of VEGFR1 reduced the migration ability of endothelial cells due to the suppression of the cell actin cytoskeleton reorganization [55].

For L1/anti-endoglin polyplexes, we formed complexes at the N/P ratios of 8/1 with 100 nM and 200 nM siRNA, and 16/1 with 100 nM siRNA (Figure 5c). L1/anti-endoglin 16/1 complexes with 200 nM siRNA were excluded from the study because of their cytotoxicity (Figure 1c). Significant differences in the migration activity after the cell treatment with anti-endoglin siRNA and mock siRNA-complexes were demonstrated only when 200 nM siRNA concentrations were used. The decrease of cell migration was found to be up to 57% compared to mock siRNA-complexes. Previously, it has been demonstrated that RNAi-based inhibition of endoglin gene expression in human and mouse endothelial cells decreased their migration potential [44]. However, in a number of studies, this effect was found only after TGF-β addition into the culture medium [56]. Moreover, Lee and colleagues demonstrated an opposite effect in endoglin-negative mouse embryonic endothelial cells, which had a higher migration ability compared to wild-type cells [57]. Thus, the role of endoglin in angiogenesis should be elucidated in further studies.

In order to reveal a possible synergistic effect on endothelial cells migration, several siRNAs were co-delivered by L1-based polyplexes (Figure 5d). Combinatorial delivery of several siRNAs may affect the regulatory functions of the cellular miRNAs due to selective incorporation into a RISC complex [58]. However, the optimization of siRNA concentrations can weaken the competition with the miRNAs [59]. Anti-VEGFR1 or anti-endoglin siRNAs were combined with anti-VEGFA siRNA with a final concentration of 200 nM and complexed with L1 at a N/P ratio of 8/1. We did not use triple siRNA polyplexes because of cytotoxicity that could be induced by a high total concentration of siRNA (Figure 1c). Combined anti-VEGFA+anti-VEGFR1 and anti-VEGFA+anti-endoglin siRNA polyplexes decreased the relative number of migrated cells up to 52% and 47%, respectively, in comparison with control mock siRNA-complexes (Figure 5d). We did not observe acumulative effect from the combined anti-VEGFA+anti-VEGFR1 siRNA delivery. In fact, the migration suppression efficiency of anti-VEGFR1 siRNA-polyplexes alone was equal to that of combined anti-VEGFA+anti-VEGFR1 siRNA-polyplexes. In contrast, the synergistic effect of anti-VEGFA+anti-endoglin siRNA co-delivery was demonstrated and significant differences between the efficiency of these complexes and anti-VEGFA siRNA-polyplexes treatments were found (*p* < 0.05) (Figure 5d). Also, it should be noted that 100 nM anti-endoglin siRNA-polyplexes treatment did not reduce the migration of the endothelial cells (Figure 5c). The synergistic effect of combinatorial RNAi knockdown was already described in several works devoted to cancer gene therapy. For example, combinatorial siRNA targeting of EGF-Receptor and Akt2 induced tumor specific apoptosis and significantly increased survival in intracerebral glioblastoma mouse models [60]. Recently, Kamaruzman and colleagues also demonstrated the synergistic effect of combinatorial siRNA targeting both growth factor receptor and anti-apoptotic genes for therapy against breast cancer [61].

### 3.5. Inhibition of the Endothelial Cells Proliferation

**Figure 6:**
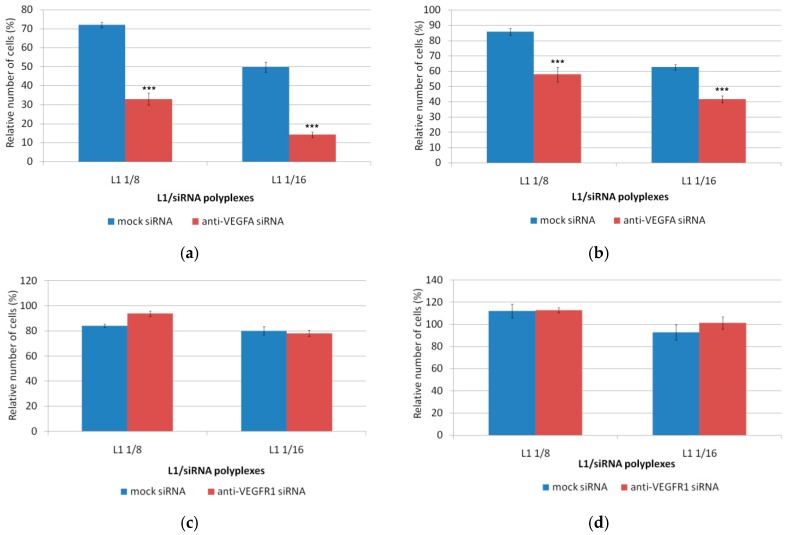

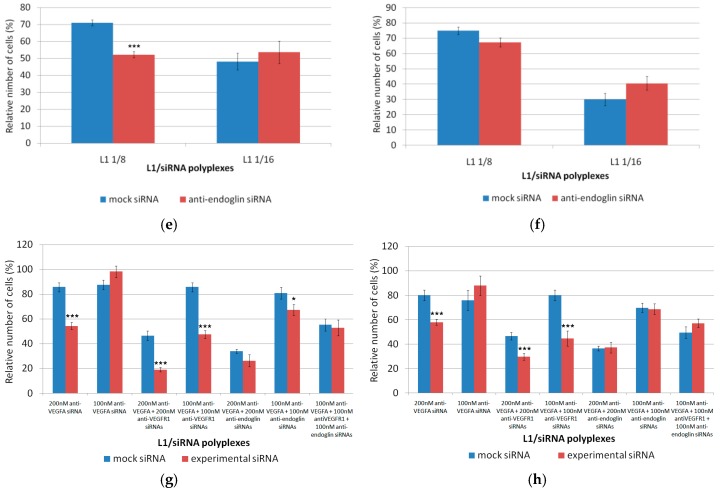
Number of EA.Hy926 cells after treatment with L1/anti-VEGFA siRNA (**a**,**b**), L1/anti-VEGFR1 siRNA (**c**,**d**), L1/ anti-endoglin siRNA (**e**,**f**), and L1/mixed siRNA complexes (**g**,**h**) analyzed using Alamar blue assay (**a**,**c**,**e**,**g**) and by crystal violet assay (**b**,**d**,**f**,**h**); * *p* < 0.05, *** *p* < 0.001 when compared with cells treated using mock siRNA.

Endothelial cells migrated from already existing blood vessels to angiogenic foci begin to proliferate under pro-angiogenic factors [62]. We used Alamar blue assay and crystal violet assay to determine whether EA.Hy926 cells proliferation was affected by anti-VEGFA, anti-VEGFR1, or anti-endoglin siRNA delivery and co-delivery. Mock siRNA-polyplexes were used as a negative control. EA.Hy926 cells were transfected with siRNA-polyplexes formed at 8/1 and 16/1 charge ratios and the siRNA concentration was 200 nM (Figure 6). It should be noted that EA.Hy926 cells were seeded at the low density in order to avoid the contact inhibition of dividing cells during the 72 h period of the proliferation assessment. Absence of the carrier-associated cytotoxicity and the successful GFP gene expression silencing after siRNA treatment of MDA-MB-231 cells seeded at the same density allowed us to perform RNAi followed by the proliferation test (Figure 1 and Figure 2). According to the results of the proliferation tests, significant differences in the cell number after the incubation period were found between the anti-VEGFA siRNA-polyplexes and mock siRNA-polyplexes. The relative cell number after the treatment with the anti-VEGFA polyplexes at siRNA concentration of 200 nM was decreased by up to 45% and 30% from that of mock siRNA, respectively, according to the Alamar blue assay data, and up to 68% and 67%, respectively, according to crystal violet assay data (Figure 6a,b). Actually, a significant decrease in the cell proliferation was expected because VEGFA plays a critical role in the angiogenesis regulation and is important for endothelial cell physiology [63]. However, it should be noted that at a lesser anti-VEGFA siRNA concentration (100 nM), the decrease in proliferation was not observed (Figure 6g,h). This fact highlights the importance of siRNA concentration optimization to obtain VEGFA gene silencing. According to Figure 6c,d, anti-VEGFR1 siRNA polyplexes did not contribute to the decrease of endothelial cell proliferation. Differences in the relative number of EA.Hy926 cells were not observed after cell treatment, both with experimental and mock siRNA-polyplexes (Figure 6c,d). Previously, it has been demonstrated that VEGFR1 activation did not lead to the proliferation of the primary endothelial cells [64]. Also, VEGFR1 suppression did not affect the VEGFA-induced HUVEC cell line proliferation [55]. Results of the Alamar blue assay showed that in the case of L1/anti-endoglin polyplexes formed at the N/P ratio of 8/1, a significant decrease in relative cell number after the anti-endoglin siRNA treatment was found (Figure 6e). However, when the crystal violet assay was used, no differences were observed (Figure 6f). On the other hand, anti-endoglin siRNA complexes formed at the 16/1 N/P ratio did not contribute to a decrease in the proliferation of endothelial cells compared with mock siRNA-polyplexes (Figure 6e,f). Dolinsek and colleagues previously demonstrated the inhibition of endothelial cell proliferation after endoglin gene expression was suppressed by RNAi [44]. Opposite results were obtained by Pan and colleagues [65]. They showed that the level of endothelial cell proliferation in endoglin negative cells was higher than that of wild-type cells. The authors explain these results by considering TGF-β-independent signaling cascades.

Previously, it was shown that the administration of drugs that inhibit angiogenesis through only VEGFA targeting results in the elevation of VEGFR1 ligands (e.g., PlGF and VEGF-B) and eventually leads to an adaptive response and drug resistance [19]. Thus, additional targeting of an alternative angiogenesis pathway could have a greater effect on angiogenesis inhibition. As both VEGFR1 and endoglin signaling modulates angiogenesis, we investigated whether targeting both receptors and VEGFA expression might result in synergistic anti-angiogenic treatment effects in EA.Hy926 endothelial cells. To determine the synergistic effects in downregulation of the endothelial cell proliferation, several siRNAs were combined in the L1-polyplexes at an 8/1 charge ratio (Figure 6g,h). The polyplexes formed at a 16/1 charge ratio were excluded from the cell proliferation study because of an absence of difference between the two formulations in RNAi efficacy (Figure 2 and Figure 3) and to avoid possible toxic effects of 16/1 polyplexes found in cytotoxicity experiments (Figure 1b). Anti-VEGFR1 or anti-endoglin siRNAs were mixed with anti-VEGFA siRNA at equimolar concentrations of 100 nM and 200 nM of both molecules. These polyplexes were compared with anti-VEGFA siRNA alone complexed by L1 at the concentration of 100 nM and 200 nM, respectively. We also studied triple siRNA-polyplexes consisted of L1 and 100 nM of anti-VEGFR1, anti-endoglin, and anti-VEGFA siRNAs. Similarly, the efficiency of triple combinatorial siRNA-polyplexes was compared with anti-VEGFA siRNA-polyplexes alone.

We found that the endothelial cell treatment using L1-polyplexes formed with anti-VEGFA and anti-VEGFR1 siRNAs at a concentration of 200 nM resulted in a decrease of cell proliferation up to 42% according to the Alamar blue assay results. Respective anti-VEGFA siRNA-polyplexes were less effective and decreased the cell proliferation up to 64% at the same siRNA concentration (Figure 6g). Similarly, treatment with L1-polyplexes formed with anti-VEGFA and anti-VEGFR1 siRNAs at a concentration of 100 nM, which led to a decrease of proliferation up to 55% (Figure 6g). In contrast, treatment with anti-VEGFA siRNA-polyplexes at the same siRNA concentration did not inhibit cell proliferation and was not significantly different from treatment with mock siRNA-polyplexes. Alamar blue assay results were concordant with crystal violet assay data (Figure 6h).

Thus, the combination of anti-VEGFA and anti-VEGFR1 siRNA resulted in a 1.5-fold increase of anti-angiogenic properties of L1-based polyplexes (*p* < 0.001) (Figure 6g,h). The results obtained clearly show that the combinatorial down-regulation of two VEGF-pathways led to a synergistic anti-angiogenic effect confirmed via migration and proliferation studies on the endothelial cells. Previously, it was demonstrated that combinatorial co-delivery of siRNAs against several targets could be a powerful approach to treat cancer [59,66,67]. Two strategies of combinatorial siRNA delivery were proposed. The first was copolymerization of two different siRNA sequences in a single backbone of siRNA polymer [66,67] and the second was the formation of combined siRNA-polyplexes via simultaneous complexation of multiple siRNAs with non-viral carriers [59]. Both strategies of combinatorial treatment led to synergistic anti-cancer and anti-angiogenic effects. On the over hand, combinatorial anti-VEGFA+anti-endoglin siRNAs-polyplexes and triple siRNA polyplexes mostly did not contribute to a decrease of the cell proliferation. We found that only treatment with L1-polyplexes formed with anti-VEGFA and anti-endoglin siRNAs at 100 nM concentration resulted in a slight decrease of proliferation up to 16% when analyzed using the Alamar blue assay (Figure 6g). However, this was not confirmed by the crystal violet analysis (Figure 6h).

The results of simultaneous VEGFA and endoglin suppression may be explained by considering the opposing functional activities of endoglin isoforms. Two main isoforms, membrane-anchored long endoglin (CD105-L) and soluble short endoglin (CD105-S), differ from each other regarding their cytoplasmic tails and functions [68]. CD105-L has been shown to be proangiogenic, while CD105-S exerts the opposite effect [69]. Previously, it was found that soluble CD105-S can bind some members of the TGF-β superfamily, preventing their interaction with CD105-L, and thus eventually down-regulating angiogenesis [24].The obtained results are consistent with that of Pan and colleagues who demonstrated that TGF-β-independent signaling cascades that could adversely affect cell proliferation after endoglin suppression [65]. Triple siRNA-based silencing of VEGFA, VEGFR1, and endoglin genes expression abolished anti-proliferative properties of L1-polyplexes (Figure 5g,h). The most likely explanation of this fact is the involvement of non-VEGF pro-angiogenic pathways (e.g., FGF signaling) that could be activated after significant down-regulation of the main pro-angiogenic factors (VEGFA, VEGFR1) and modulators (Endoglin) [70]. Furthermore, important information can be acquired from our data that is essential for the future development of combinatorial anti-angiogenic treatment (Table 1). According to the obtained results, the simultaneous silencing of several pro-angiogenic pathways may not be always beneficial for the efficiency of anti-angiogenic therapy as it was demonstrated by the endothelial cell treatment with triple siRNAs L1-polyplexes.

## 4. Conclusions

Targeting of angiogenesis by means of RNA interference is a promising and highly necessary approach for treatment of angiogenesis-related diseases induced by abnormally stimulated neovascularization such as cancer, atherosclerosis, age-related macular degeneration, endometriosis, etc. However, the need for efficient and specific siRNA delivery systems is of importance for the development of this promising approach. The efficiency of an anti-angiogenic treatment can be further improved via the combinatorial delivery of siRNAs against multiple pro-angiogenic targets. Here, we have demonstrated the efficient down-regulation of endothelial cells migration and proliferation by anti-VEGFA, anti-VEGFR1, and anti-Endoglin siRNA delivery mediated by peptide-based vector L1. Several types of single and combinatorial L1-based siRNA polyplexes have been studied and the most efficient formulation has been found. Based on our findings, we have concluded that a combinatorial treatment by L1-polyplexes formed with anti-VEGFA and anti-VEGFR1 siRNAs effectively inhibits migration and proliferation of endothelial cells and can be suggested as a useful tool for anti-angiogenic therapy.

## Figures and Tables

**Table 1:** Summary of registered anti-angiogenic effects after anti-VEGFA, anti-VEGFR1, or anti-endoglin siRNA delivery and co-delivery mediated by an L1 vector (↓ means decrease of migration/proliferation; − means no effect; ↓/− means effect detected only by Alamar Blue assay; ↓↓ means synergistic effect; NA means absence of data due to cytotoxicity).

Type of Analysis	Type of siRNA					
	Anti-VEGFA	Anti-VEGFR1	Anti-Endoglin	Anti-VEGFA + Anti-VEGFR1	Anti-VEGFA + Anti-Endoglin	Anti-VEGFA + Anti-VEGFR1 + Anti-Endoglin
Migration	↓	↓	↓	↓	↓↓	NA
Proliferation	↓	−	↓/−	↓↓	−	−

## References

[1] Park C., Kim T.M., Malik A.B. (2013). Transcriptional regulation of endothelial cell and vascular development. Circ. Res..

[2] Ferrara N., Kerbel R.S. (2005). Angiogenesis as a therapeutic target. Nature.

[3] Gacche R.N., Meshram R.J. (2014). Angiogenic factors as potential drug target: Efficacy and limitations of anti-angiogenic therapy. Biochim. Et Biophys. Acta—Rev. Cancer.

[4] Bennett M.R., Sinha S., Owens G.K. (2016). Vascular Smooth Muscle Cells in Atherosclerosis. Circ. Res..

[5] Kerbel R., Folkman J. (2002). Clinical translation of angiogenesis inhibitors. Nat. Rev. Cancer.

[6] Shubina A.N., Egorova A.A., Baranov V.S., Kiselev A.V. (2013). Recent advances in gene therapy of endometriosis. Recent Pat. Dna Gene Seq..

[7] Li T., Kang G., Wang T., Huang H.E. (2018). Tumor angiogenesis and anti-angiogenic gene therapy for cancer. Oncol. Lett..

[8] Ferrara N. (2001). Role of vascular endothelial growth factor in regulation of physiological angiogenesis. Am.J.Physiol.—CellPhysiol..

[9] Behrouz R., Malek A.R., Torbey M.T. (2012). Small vessel cerebrovascular disease: The past, present, and future. StrokeRes.Treat..

[10] Gaengel K., Genové G., Armulik A., Betsholtz C. (2009). Endothelial-mural cell signaling in vascular development and angiogenesis. Arterioscler. Thromb. Vasc. Boil..

[11] Liu S., Kong X., Ge D., Wang S., Zhao J., Su L., Zhang S., Zhao B., Miao J. (2016). Identification of new small molecules as apoptosis inhibitors in vascular endothelial cells. J. Cardiovasc. Pharmacol..

[12] Gerber H.P., Hillan K.J., Ryan A.M., Kowalski J., Keller G.A., Rangell L., Wright B.D., Radtke F., Aguet M., Ferrara N. (1999). VEGF is required for growth and survival in neonatal mice. Development.

[13] Safran M., Kaelin W.G. (2003). HIF hydroxylation and the mammalian oxygen-sensing pathway. J. Clin. Investig..

[14] Morfoisse F., Renaud E., Hantelys F., Prats A.-C., Garmy-Susini B. (2015). Role of hypoxia and vascular endothelial growth factors in lymphangiogenesis. Mol.Cell.Oncol..

[15] Luo H., Li B., Li Z., Cutler S.J., Rankin G.O., Chen Y.C. (2013). Chaetoglobosin K inhibits tumor angiogenesis through downregulation of vascular epithelial growth factor-binding hypoxia-inducible factor 1α. Anti-Cancer Drugs.

[16] Fischer C., Mazzone M., Jonckx B., Carmeliet P. (2008). FLT1 and its ligands VEGFB and PlGF: Drug targets for anti-angiogenic therapy?. Nat. Rev. Cancer.

[17] Kami J., Muranaka K., Yanagi Y., Obata R., Tamaki Y., Shibuya M. (2008). Inhibition of choroidal neovascularization by blocking vascular endothelial growth factor receptor tyrosine kinase. Jpn. J. Ophthalmol..

[18] Golfmann K., Meder L., Koker M., Volz C., Borchmann S., Tharun L., Dietlein F., Malchers F., Florin A., Büttner R. (2018). Synergistic anti-angiogenic treatment effects by dual FGFR1 and VEGFR1 inhibition in FGFR1-amplified breast cancer. Oncogene.

[19] Cao Y. (2009). Positive and negative modulation of angiogenesis by VEGFR1 ligands. Sci.Signal..

[20] Derbyshire E.J., Gazdar A.F., King S.W., Thorpe P.E., Derbyshire E.J., King S.W., Thorpe P.E., Gazdar A.F., Vitetta E.S., Tazzari P.L. (1995). Up-Regulation of Endoglin on Vascular Endothelial Cells in Human Solid Tumors: Implications for Diagnosis and Therapy. Clin. Cancer Res..

[21] Nassiri F., Cusimano M.D., Scheithauer B.W., Rotondo F., Fazio A., Yousef G.M., Syro L.V., Kovacs K., Lloyd R.V. (2011). Endoglin (CD105): A review of its role in angiogenesis and tumor diagnosis, progression and therapy. Anticancer Res..

[22] Li D.Y., Sorensen L.K., Brooke B.S., Urness L.D., Davis E.C., Taylor D.G., Boak B.B., Wendel D.P. (1999). Defective angiogenesis in mice lacking endoglin. Science.

[23] Taskiran C., Erdem O., Onan A., Arisoy O., Acar A., Vural C., Erdem M., Ataoglu O., Guner H. (2006). The prognostic value of endoglin (CD105) expression in ovarian carcinoma. Int. J. Gynecol. Cancer.

[24] Kasprzak A., Adamek A. (2018). Role of Endoglin (CD105) in the Progression of Hepatocellular Carcinoma and Anti-Angiogenic Therapy. Int. J. Mol. Sci..

[25] Seon B.K., Haba A., Matsuno F., Norihiko Takahashi M.T., She X., Harada N., Uneda S., Tsujie T., Toi H., Hilda Tsai Y.H. (2011). Endoglin-targeted cancer therapy. Curr. Drug Deliv..

[26] Smirnov I.V., Gryazeva I.V., Samoilovich M.P., Klimovich V.B. (2015). Endoglin (CD105) - A target for visualization and anti-angiogenic therapy for malignant tumors. Vopr. Onkol..

[27] Elbashir S.M., Harborth J., Lendeckel W., Yalcin A., Weber K., Tuschl T. (2001). Duplexes of 21-nucleotide RNAs mediate RNA interference in cultured mammalian cells. Nature.

[28] David S., Pitard B., Benoît J.P., Passirani C. (2010). Non-viral nanosystems for systemic siRNA delivery. Pharm. Res..

[29] Le Bon B., Van Craynest N., Daoudi J.M., Di Giorgio C., Domb A.J., Vierling P. (2004). AMD3100 Conjugates as Components of Targeted Nonviral Gene Delivery Systems: Synthesis and in Vitro Transfection Efficiency of CXCR4-Expressing Cells. Bioconjug. Chem..

[30] Driessen W.H.P., Fujii N., Tamamura H., Sullivan S.M. (2008). Development of peptide-targeted lipoplexes to CXCR4-expressing rat glioma cells and rat proliferating endothelial cells. Mol. Ther..

[31] Egorova A., Kiselev A., Hakli M., Ruponen M., Baranov V., Urtti A. (2009). Chemokine-derived peptides as carriers for gene delivery to CXCR4 expressing cells. J. Gene Med..

[32] Wang Y., Xie Y., Oupický D. (2016). Potential of CXCR4 / CXCL12 Chemokine Axis in Cancer Drug Delivery. Curr. Pharmacol. Rep..

[33] Juarez J., Bendall L., Bradstock K. (2005). Chemokines and their Receptors as Therapeutic Targets: The Role of the SDF-1 / CXCR4 Axis. Curr. Pharm. Des..

[34] Salcedo R., Oppenheim J.J. (2003). Role of chemokines in angiogenesis: CXCL12/SDF-1 and CXCR4 interaction, a key regulator of endothelial cell responses. Microcirculation.

[35] Kiselev A., Egorova A., Laukkanen A., Baranov V., Urtti A. (2013). Characterization of reducible peptide oligomers as carriers for gene delivery. Int. J. Pharm..

[36] Egorova A., Bogacheva M., Shubina A., Baranov V., Kiselev A. (2014). Development of a receptor-targeted gene delivery system using CXCR4 ligand-conjugated cross-linking peptides. J.GeneMed..

[37] Egorova A., Shubina A., Sokolov D., Selkov S., Baranov V., Kiselev A. (2016). CXCR4-targeted modular peptide carriers for efficient anti-VEGF siRNA delivery. Int.J.Pharm..

[38] Egorova A., Petrosyan M., Maretina M., Balashova N., Polyanskih L., Baranov V., Kiselev A. (2018). Anti-angiogenic treatment of endometriosis via anti-VEGFA siRNA delivery by means of peptide-based carrier in a rat subcutaneous model. Gene Ther..

[39] Egorova A.A., Maretina M.A., Kiselev A.V. (2019). VEGFA Gene Silencing in CXCR4-Expressing Cells via siRNA Delivery by Means of Targeted Peptide Carrier. Methods Mol. Biol..

[40] Slita A., Egorova A., Casals E., Kiselev A., Rosenholm J.M. (2018). Characterization of modified mesoporous silica nanoparticles as vectors for siRNA delivery. Asian J. Pharm. Sci..

[41] Edgell C.J.S., McDonald C.C., Graham J.B. (1983). Permanent cell line expressing human factor VIII-related antigen established by hybridization. Proc. Natl. Acad. Sci. USA.

[42] De Fougerolles A., Frank-Kamenetsky M., Manoharan M., Rajeev K.G., Hadwiger P. (2011). IRNA Agents Targeting VEGF, U.S. Patent No. 7,919,473.

[43] Zhou Z., Zhao C., Wang L., Cao X., Li J., Huang R., Lao Q., Yu H., Li Y., Du H. (2015). A VEGFR1 antagonistic peptide inhibits tumor growth and metastasis through VEGFR1-PI3K-AKT signaling pathway inhibition. Am. J. Cancer Res..

[44] Dolinsek T., Markelc B., Bosnjak M., Blagus T., Prosen L., Kranjc S., Stimac M., Lampreht U., Sersa G., Cemazar M. (2015). Endoglin silencing has significant antitumor effect on murine mammary adenocarcinoma mediated by vascular targeted effect. Curr.GeneTher..

[45] Raemdonck K., Naeye B., Høgset A., Demeester J., De Smedt S.C. (2010). Prolonged gene silencing by combining siRNA nanogels and photochemical internalization. J.Control.Release.

[46] Wu J., Qu L., Meng L., Zeng Y., Shou C., Xu H., Jiang B., Ren T. (2012). N -α-Acetyltransferase 10 protein inhibits apoptosis through RelA/p65-regulated MCL1 expression. Carcinogenesis.

[47] Whitehead K.A., Langer R., Anderson D.G. (2009). Knocking down barriers: advances in siRNA delivery. Nat.Rev.Drug Discov..

[48] Müller A., Homey B., Soto H., Ge N., Catron D., Buchanan M.E., McClanahan T., Murphy E., Yuan W., Wagner S.N. (2001). Involvement of chemokine receptors in breast cancer metastasis. Nature.

[49] Wang Z., Ma Y., Yu X., Niu Q., Han Z., Wang H., Li T. (2018). Targeting CXCR4 – CXCL12 Axis for Visualizing, Predicting, and Inhibiting Breast Cancer Metastasis with Theranostic AMD3100 – Ag2S Quantum Dot Probe. Adv. Funct. Mater..

[50] Vega-Villa K.R., Takemoto J.K., Yáñez J.A., Remsberg C.M., Forrest M.L., Davies N.M. (2008). Clinical toxicities of nanocarrier systems. Adv. Drug Deliv. Rev..

[51] Vaidyanathan S., Anderson K.B., Merzel R.L., Jacobovitz B., Kaushik M.P., Kelly C.N., Van Dongen M.A., Dougherty C.A., Orr B.G., Banaszak Holl M.M. (2015). Quantitative Measurement of Cationic Polymer Vector and Polymer-pDNA Polyplex Intercalation into the Cell Plasma Membrane. ACS Nano.

[52] Jones N.A., Hill I.R.C., Stolnik S., Bignotti F., Davis S.S., Garnett M.C. (2000). Polymer chemical structure is a key determinant of physicochemical and colloidal properties of polymer–DNA complexes for gene delivery. Biochim. Biophys. Acta—Gene Struct. Expr..

[53] Herbert S.P., Stainier D.Y.R. (2011). Molecular control of endothelial cell behaviour during blood vessel morphogenesis. Nat. Rev. Mol. Cell Biol..

[54] Herzog B., Pellet-Many C., Britton G., Hartzoulakis B., Zachary I.C. (2011). VEGF binding to NRP1 is essential for VEGF stimulation of endothelial cell migration, complex formation between NRP1 and VEGFR2, and signaling via FAK Tyr407 phosphorylation. Mol. Boil. Cell.

[55] Kanno S., Oda N., Abe M., Terai Y., Ito M., Shitara K., Tabayashi K., Shibuya M., Sato Y. (2000). Roles of two VEGF receptors, Flt-1 and KDR, in the signal transduction of VEGF effects in human vascular endothelial cells. Oncogene.

[56] Warrington K., Hillarby M.C., Li C., Letarte M., Kumar S. (2005). Functional role of CD105 in TGF-β1 signalling in murine and human endothelial cells. Anticancer Res..

[57] Lee N.Y., Ray B., How T., Blobe G.C. (2008). Endoglin promotes transforming growth factor β-mediated Smad 1/5/8 signaling and inhibits endothelial cell migration through its association with GIPC. J. Biol. Chem..

[58] Castanotto D., Sakurai K., Lingeman R., Li H., Shively L., Aagaard L., Soifer H., Gatignol A., Riggs A., Rossi J.J. (2007). Combinatorial delivery of small interfering RNAs reduces RNAi efficacy by selective incorporation into RISC. Nucleic Acids Res..

[59] Tiash S., Kamaruzman N.I.B., Chowdhury E.H. (2017). Carbonate apatite nanoparticles carry siRNA(S) targeting growth factor receptor genes egfr1 and erbb2 to regress mouse breast tumor. Drug Deliv..

[60] Michiue H., Eguchi A., Scadeng M., Dowdy S.F. (2009). Induction of in vivo synthetic lethal RNAi responses to treat glioblastoma. Cancer Biol..

[61] Kamaruzman N., Tiash S., Ashaie M., Chowdhury E. (2018). siRNAs Targeting Growth Factor Receptor and Anti-Apoptotic Genes Synergistically Kill Breast Cancer Cells through Inhibition of MAPK and PI-3 Kinase Pathways. Biomedicines.

[62] Carmeliet P., Jain R.K. (2011). Molecular mechanisms and clinical applications of angiogenesis. Nature.

[63] Kaufmann P., Mayhew T.M., Charnock-Jones D.S. (2004). Aspects of human fetoplacental vasculogenesis and angiogenesis. II. Changes during normal pregnancy. Placenta.

[64] Seetharam L., Gotoh N., Maru Y., Neufeld G., Yamaguchi S., Shibuya M. (1995). A unique signal transduction from FLT tyrosine kinase, a receptor for vascular endothelial growth factor VEGF. Oncogene.

[65] Pan C.C., Bloodworth J.C., Mythreye K., Lee N.Y. (2012). Endoglin inhibits ERK-induced c-Myc and cyclin D1 expression to impede endothelial cell proliferation. Biochem. Biophys. Res. Commun..

[66] Lee S.J., Yook S., Yhee J.Y., Yoon H.Y., Kim M.G., Ku S.H., Kim S.H., Park J.H., Jeong J.H., Kwon I.C. (2015). Co-delivery of VEGF and Bcl-2 dual-targeted siRNA polymer using a single nanoparticle for synergistic anti-cancer effects in vivo. J. Control. Release.

[67] Jang M., Han H.D., Ahn H.J. (2016). A RNA nanotechnology platform for a simultaneous two-in-one siRNA delivery and its application in synergistic RNAi therapy. Sci. Rep..

[68] Hu J., Guan W., Liu P., Dai J., Tang K., Xiao H., Qian Y., Sharrow A.C., Ye Z., Wu L. (2017). Endoglin Is Essential for the Maintenance of Self-Renewal and Chemoresistance in Renal Cancer Stem Cells. Stem Cell Rep..

[69] Pérez-Gómez E., Eleno N., López-Novoa J.M., Ramirez J.R., Velasco B., Letarte M., Bernabéu C., Quintanilla M. (2005). Characterization of murine S-endoglin isoform and its effects on tumor development. Oncogene.

[70] Clarke J.M., Hurwitz H.I. (2013). Understanding and targeting resistance to anti-angiogenic therapies. J. Gastrointest. Oncol..

